# The Sustainability of the Fishery Industry and Environmental Development: A Study on Factor Market Distortions

**DOI:** 10.3390/ijerph20043017

**Published:** 2023-02-09

**Authors:** Sha Yang, Jia Wu

**Affiliations:** School of Business, Ningbo University, Ningbo 315211, China

**Keywords:** marine fishery, industrial structure adjustment, factor market distortion, qualitative comparative analysis

## Abstract

By reviewing the related research on the distortion of labor, capital, and technical factors, combined with the development and the upgrading status of the marine fishery industry, we used the macro data of the industry to measure the degree of price distortion of its market factors and to construct a Moore-like index and a simplified industrial structure upgrade index based on the fsQCA fuzzy set qualitative comparative analysis. The main content of this paper is related to environment and sustainable development. We found that (1) in the case of low capital factor distortion, the combination of high labor factor distortion and low marine fishery resource distortion will inhibit the rapid upgrading of the marine fishery industry structure; (2) in the case of low capital factor distortion, the combination of low labor factor distortion and high marine fishery resources will also inhibit the rapid upgrading of the marine fishery industry structure; and (3) under the combination of low labor factor distortion and low marine fishery resource factor distortion, regardless of the degree of capital factor distortion, the rapid upgrading of the marine fishery industrial structure will be inhibited; there are only differences in the timing of the impact. The impact of factor distortion on the upgrading of industrial structure lags two and three periods, respectively.

## 1. Introduction

Marine fishery is an important component of the marine industry, and it has an important impact on the economic and social development of coastal areas [1], involved in marine environment and sustainable development. For a long time, the marine fishery industry has had a single structure, and it has remained at a primary stage for a long time. The fluctuation range of the vulnerability of the industrial ecosystem has gradually increased, and the coastal areas are facing comprehensive problems of sustainable development [2]. With the limited reproduction and regeneration capacity of its marine fishery resources, coastal waters show the trend of overfishing, a decline in fishery resources, and the deterioration of its coastal ecological environment [3]. The desertification of fishing grounds, such as the disappearance of offshore fishing seasons and even the lack of fish due to overfishing and habitat destruction, has become a focus [4]. Fishing technology, fishing intensity, and fishery industry structure have gradually become important factors that affect the sustainable utilization of marine fishery resources [5].

As the fishery industry has developed, the primary industry has always accounted for an absolute proportion, amounting to about 50% of the total output value. The secondary industry and the tertiary industry have similar development levels. The overall economic efficiency of marine fishery is low, and its development speed is relatively slow [6]. The rationalization of the marine fishery industry structure has a significant “smoothing effect” on the marine fishery economic fluctuations [7]. It is dominated by the primary industry; the development of the secondary and tertiary industries is lagging. The situation is urgent, and it needs to be improved. As it changes from the current extensive mode of relying on the high input of factors to the new intensive mode driven by efficiency, giving full play to resource endowment and improving the efficiency of the fishery economy is the key direction for fishery development in the future [8]. How environmental regulation affects factor allocation is becoming an emerging hot topic [9]. Government regulation has a positive and significant impact on the green production behavior of the marine fishery industry [10], and it often uses environmental policy to induce green investment [11]. Although a series of special measures for the governance of offshore fishery resources have been successively introduced, on the whole, this has not solved the long-standing imbalance between “too many input elements” and “too much output.” The unreasonable structure of the fishery industry is inseparably related to the distortion of the factor market. The distortion of the factor market makes it difficult for factors to be freely allocated among various industries according to market laws, and this directly leads to the Pareto inefficiency of factor allocation [12,13]. There is a significant negative impact on the improvement of production efficiency [13,14], and different industries have different benefits from factor distortion.

In the existing literature, the research on factor market distortion is basically carried out from the perspective of labor factors, capital factors, and technological factors. In terms of the labor factor market, under the ideal conditions of a perfectly competitive market, the equilibrium wage of laborers is determined by the marginal productivity of labor [15], but in reality, the two may be unequal, resulting in labor market distortions. In the capital factor market, when the benchmark interest rate deviates from the market interest rate, the market is considered to be distorted. In terms of technical efficiency, innovative research and development as well as technological progress have a considerable impact on the productivity of enterprises [16]. The improvement of technical efficiency has led to the growth of the total factor productivity, and this is the main reason for the productivity differences across regions [17].

Most of the existing studies focus on the discussion of capital, land, and labor factors and focus less on natural resources and institutional factors—and even less on the impact on the internal structure of a certain industry. There are few studies on the impact mechanism and action path of the adjustment of the marine fishery industry structure based on the perspective of factor market distortion. Some relevant studies have the following problems: first, the research perspective is focused on the impact of a single factor or several factors on the development of marine fisheries; second, the research on the relationship between factor input and the optimal development of marine fisheries mostly stays at the level of theoretical analysis, while the research on the direct relationship between factor market distortion and the industrial structure of marine fisheries is less studied, especially the quantitative and empirical research are lacking; third, it is necessary to attach importance to the research on the increase of the quantity or quality of the factors, and pay little attention to the impact of the factor allocation effect on the industrial development.

Since it is based on the current development and upgrading of the marine fishery industry, this paper uses industry macro data to measure the degree of market factor price distortion, and it constructs a Moore-like index and a simplified industrial structure upgrade index. Based on the qualitative comparative analysis of fsQCA fuzzy sets, this paper studies the impact of factor market distortion on the fishery industry structure. An examination of the influence of factor distortion on industrial structure upgrading under different circumstances is a helpful tool in better understanding the influence of factor distortion on industrial structure and in providing a reference for how to carry out the market-oriented reform of factors in order to promote the development of industrial structure.

The rest of this paper proposes the analysis method and a logical framework that measures the distortion degree of marine fishery industry factors and an industrial structure index, analyzes the impact of factor market distortion on the fishery industry structure by using the qualitative comparative analysis method of fsQCA, and explains the conclusions of the fsQCA analysis based on the current situation of factor market distortion in the marine fishery industry. The paper finally summarizes and puts forward relevant countermeasures and suggestions.

The possible marginal contribution of this paper is that the analysis and selection of factor market distortion in the existing research are mostly limited to the two factors of capital and labor. In combination with the actual situation of the marine fishery industry, we have expanded the interpretation scope of factor market distortion and revealed the micro mechanism and macro transmission mechanism of the relationship between factor market distortion and the optimization and upgrading of the marine fishery industry from the theoretical level. We have explored and constructed an index model to comprehensively evaluate the market distortion of marine fishery factors and the optimization and upgrading of the marine fishery industry, which provides effective support for subsequent research and analysis.

## 2. Research Methods

### 2.1. Analysis Method and Logical Framework

The degree of market distortion of various factors in this study is affected by factors such as macroeconomics and industrial policies, and there is a possibility of interdependence. The causal relationship between it and the adjustment of the marine fishery industry structure is non-linear. At the same time, the degree of distortion of each element and the measurement of the structural status of the marine fishery industry are both fuzzy estimates rather than accurate measurements; this conforms to the requirements of the real world and to objective laws [18].

Based on the limitation of symmetrical thinking in the traditional quantitative analysis of the correlation coefficient between cause and effect, in symmetrical analysis, the direction between a single cause and effect depends on other conditions in the given environment [19], but the interaction between multiple explanatory perspectives is often ignored [20]. The mindset of using models for validation likewise ignores the obvious fact that the same result can be reached by multiple paths.

In view of the above limitations, this paper introduces the method of QCA (qualitative comparative analysis), both to study the joint effect of the market distortion of the above three factors on the adjustment of the marine fishery industry structure and to explore the possible interaction effects between the different factors. The QCA method is a case study-oriented theoretical set research method; it integrates analytical techniques such as sets and Boolean algebra, it integrates the advantages of qualitative and quantitative research methods, and it provides a third way beyond qualitative research and quantitative research roads [21,22].

### 2.2. Calculation of Distortion Degree of Factor Prices in Marine Fisheries Market

#### 2.2.1. Construction of Marine Fishery Production Function Model

Based on the economic growth point of view of the classical economics school, the neoclassical school of economics established a neoclassical production function [23] which is expressed as:

Y=F(K,L), (K>0, L>0)

The neoclassical production function satisfies the law of diminishing marginal returns and constant returns to scale, as well as the law that states that, as capital (or labor) tends to zero, the marginal product of capital (or labor) tends to infinity. Solow incorporates technology levels into the production function as another factor to promote economic growth and establishes a more complete production function [24], arguing that technological progress will also promote economic growth without changing the input of labor and capital factors. Its expression is:

Y=A·F(K,L)

with *A* representing the level of technological development.

The input of several different elements plays a key role in the development of a marine fishery. Considering the availability of data, this paper selects the four elements of technology level, labor force, capital, and marine fishery resources to construct a production function, and obtains the production function of a marine fishery:

(1) $$Y=F\left(L,R,K\right)=A_{i}\cdot L^{\alpha }K^{\beta }R^{\gamma }\;A_{i}=A_{0}\cdot e^{at}$$

*Y* represents the output value of marine fishery; *t* represents time; $\;A_{0}$ is a constant and represents the technical level of the base year; $A_{i}$ represents the comprehensive scientific and technological level in the year *t*; *L* represents labor force; *K* represents capital; *R* represents marine fishery resources; α, β, γ  are the elasticity coefficients of labor, capital, and marine fishery resources, respectively.

#### 2.2.2. Calculation of the Elastic Coefficient of Each Element of Marine Fishery

The logarithmic form of the C-D raw function can be expressed as:

(2) $$LnY=lnA_{0}+at+\alpha lnL+\beta lnK+\gamma lnR$$

Substitute α+β+γ=1 into Equation (2) to get:

(3) $$Ln\frac{Y}{L}=\;lnA_{0}+at+\beta ln\frac{K}{L}+\;\gamma ln\frac{R}{L}$$

Among them, Y⁄L is the per capita output value, K⁄L is the per capita capital, R⁄L is the per capita fishery resources, and $lnA_{0}+at$ is a constant. Combined with marine fishery output and input data, the ordinary least squares method is used to estimate the output elasticity of each development factor. By regression, we can get estimated values $\hat{\alpha },\;\hat{\beta },\;\hat{\gamma }$ from α, β, γ. We can calculate the marginal product *MP* of labor, capital, and fishery resources:

(4) $$MP_{K}=\hat{\beta }\frac{Y}{K}$$

(5) $$MP_{L}=\hat{\alpha }\frac{Y}{L}$$

(6) $$MP_{R}=\hat{\gamma }\frac{Y}{R}$$

$\bar{Y},\;\bar{K},\;\bar{L},\;\bar{R}$ represent the average value of *Y*, *L*, *K*, *R.*

Assuming that ω, *r*, and φ represent the actual remuneration (i.e., the actual price) obtained by the capital factor, the labor factor, and the fishery resource factor in the market, respectively, then the ratio of the marginal output of each factor to its actual remuneration Dis is the distortion coefficient of the factor, namely:

(7) $$Dis_{K}=\frac{MP_{K}}{\omega }$$

(8) $$Dis_{L}=\frac{MP_{L}}{r}$$

(9) $$Dis_{R}=\frac{MP_{R}}{\varphi }$$

### 2.3. Factor Market Distortion and Fishery Industry Structure

The heterogeneous distortion of the factors of production is the basis and the main reason for determining the dynamic changes of the industrial structure; this has been repeatedly confirmed by relevant studies. Through systematic GMM estimation, Zong and Zhou found that factor distortions inhibit industrial upgrading [25]; Zheng and Liu believed that the relative distortion of factor prices led to the lag in the evolution of industrial structure and that the distortion of capital factor markets would promote the deepening of industrial capital [26]; this is particularly obvious in tertiary industry. This has hindered the optimization and upgrading of our country’s, China’s, industrial structure; Chen believes that the distortion of labor prices has a greater impact on the industrial structure than the price distortion of capital and energy [27].

On the basis of the degree of distortion of factors in marine fishery industry as described above, we further analyze the relationship between the distortion of factors and the industrial structure. Due to the long cycle of the marine fishery industry, as we analyze the impact of factor distortion on the industrial structure, it is necessary to consider its “hysteresis” when studying the effect of factor distortion in the t period on the industry in the *t* + 1, *t* + 2, and *t* + 3 periods, respectively.

In the research related to industrial structure, the Moore index, proposed by Moore [28], has been generally recognized. The industrial structure upgrading index, proposed by Xu [29], has also been widely used in the research. This paper refers to these two methods to measure the changes in the structure of the country’s fishery industry and to make certain adjustments and simplifications according to the actual research objects.

(1) Moore-like index

Drawing on the model of Zhang and Pu to describe the direction of industrial structure change [30], considering that the research object of this paper is the overall structure of the national marine fishery industry, it is simplified on the basis of the original Moore index model, which is called “Moore-like index”, denoted below.

The degree of transition from period *t* to period *t* + 1 is recorded as follows:

$${SMoore}_{t+1}^{i}==\sqrt{\frac{{\left(P_{t}^{\_i}\right)}^{2}+P_{t}^{i}\times P_{t+1}^{i}}{{\left(P_{t}^{\_i}\right)}^{2}+1}\times \frac{{\left(P_{t+1}^{\_i}\right)}^{2}+P_{t}^{i}\times P_{t+1}^{i}}{{\left(P_{t+1}^{\_i}\right)}^{2}+1}}$$

The value of the degree of industrial structure change between the *t* period and the *t* + 1 period is recorded as follows:

$$\Delta S\_Moore_{t+1}=\overset{3}{\underset{i=1}{\sum }}i\times P_{t}^{i}\times S\_Moore_{t+1}^{i}$$

Among them, *i* is the industry level. _i is other industries except the *i*-th industry; $P_{t}^{i}$ is the proportion of the output value of the *i*-th industry in the marine fishery industry to the total marine fishery output value.

(2) Industrial Structure Upgrading Index (Structural Upgrading)

Drawing on Xu’s research [29], the industrial structure upgrade index SU is constructed and simplified accordingly:

$$SU_{t}=\sum_{i=1}^{3}Y_{it}\times i$$

Among them, *Y* is the added value of the proportion of fishery industry *i* in phase *t*.

## 3. Results Analysis

### 3.1. Calculation Results of Factor Price Distortion

The measurement of the existence and the degree of factor distortion mainly involves output variables and factor input variables. The output variable is the output value of marine fisheries. In this paper, the input variables are the labor factors, the capital factors, and the resource factors. The data in this paper are from the China Fishery Statistical Yearbook (2000–2020), the China Fishery Yearbook (2000–2017), and the China Statistical Yearbook (2000–2020).

The output value of marine fisheries is measured by the sum of the output value of marine fishing and mariculture in the current year; the capital input of marine fisheries is represented by the input of fixed assets of marine fisheries; the labor force is measured by the sum of the number of marine fishing and marine aquaculture at the end of the year. The output value and the capital investment in the Statistical Yearbook are calculated by the nominal value of the price of the current year. In order to exclude the influence of price factors, the data are revised based on 1999. The unit root test was used to test the stationarity of the four-time series of labor, resources, capital, and marine fishery output value, and the result was a first-order, single-integrated stationary series. When conducting the ADF test, according to the SIC criterion, the lag one-period data are used. The ordinary least squares method is used for the regression of Formula (3), and the results are as follows:

(10) LnY=6.2523+0.2970LnL+0.2055LnK+0.4975LnR

The estimated values of the elastic coefficients α, β, γ of labor, capital, and resources can be obtained from Equation (10), which are 0.2970, 0.2055, and 0.4975, respectively. Taking the output elasticity of each industrial factor from 1999 to 2019 as constant, the marginal output of each factor is obtained according to Equations (4)–(6).

The capital required for the development of fishery enterprises basically comes from bank credit and government financial support. Drawing on the practice of Wang and Shi [31], we take the benchmark loan interest rate of six months to one year in each year from 1999 to 2019 as the capital price, and the average value is taken as the capital price if there are multiple changes in a year. We multiply the per capita fishery income of fishing households by the ratio of marine fishery output value to the total fishery output value as the labor force price, divide the marine fishery output value of the following year by the number of aquatic fry in the previous year as the price of aquatic fry, and use the consumer price index of 1999 as the base period for the reduction. We then calculate the distortion degree of each element according to Equations (7)–(9). If the distortion value is greater than 1, it means that the factor price has a negative distortion; if the value is equal to 1, it means that the factor price is reasonable, and there is no distortion; if it is less than 1, it means that the factor price has a positive distortion.

This analysis shows that the capital, labor, and resource elements are distorted to varying degrees and that the distortion of capital is greater than that of labor and resources, as shown in Table 1. The distortion of capital price ranges from 40–260 and, in most years, it is concentrated between 40–60; the distortion of labor force distribution ranges from 0.1–1.5, mainly concentrated in 0.2–0.4; and the distortion degree of marine fishery resources ranges from 0.4–0.8, and it is mainly concentrated in 0.4–0.5. When optimizing capital investment, we can more effectively promote the development of the marine fishery economy, followed by the labor force and marine fishery resources.

### 3.2. Analysis of Fishery Industry Structure

According to the data related to the primary, secondary, and tertiary industry structure in the “China Fishery Statistical Yearbook” (2000–2019), S_Moore and SU were calculated, respectively, and the results are shown in Table 2. The change in the primary industry of marine fisheries over the years has fluctuated more than that of the secondary and tertiary industries, ranging from 0.1971 to 0.7330. From 2010 to 2015, the change of the marine fishery primary industry has gradually accelerated; the change in the secondary industry is relatively stable, between 0.7320–0.7873; the degree of change in the tertiary industry showed a U-shaped trend, slowly declining from 2001 to 2011, and gradually increasing in 2012. As far as the industrial structure upgrading index is concerned, the larger the $SU_{t}$, the faster the growth rate of the proportion of higher-level industries; when the $SU_{t}$ is negative, this indicates that the industrial structure has degenerated.

### 3.3. Effect of Factor Market Distortion on Fishery Industrial Structure

After sorting out the relevant data of factor market distortion and the industrial structure change of the marine fishery industry in the case of lag phase I, lag phase II, and lag phase III, the fuzzy set qualitative comparative analysis method (fsQCA) is used to study and analyze the samples obtained.

#### 3.3.1. Variable Calibration and Necessity Analysis

The initial data were calibrated, and three anchor points were set, 90% of which are fully affiliated; 50% are intersection points, and 10% are not affiliated at all. The necessity of each variable was analyzed, and the results are shown in Table 3 and Table 4. The distortion of labor force, capital investment, and marine fishery resources is not a necessary condition for the change of the fishery industry’s structure in different lag periods.

#### 3.3.2. Configuration Analysis

The fsQCA software was used for truth table analysis. The samples whose original consistency was lower than 0.8 were deleted from the table, and the frequency was set to 1; the true values of the outcome variables for samples with a PRI consistency below 0.7 were set to 0. Considering the parsimonious solution and the intermediate solution comprehensively, four ways of high industrial structure change degree ($\Delta S\_Moore_{t+1}$) and six ways of low industrial structure change degree ($\sim \Delta S\_Moore_{t+1}$) are finally obtained, as shown in Table 5; four ways of high industrial structure upgrading index ($SU_{t}$) and seven ways of low industrial structure upgrading index ($\sim SU_{t}$) are obtained, as shown in Table 6. Due to the small number of condition variables, the prime implication is not considered, and all possible constituent paths are retained; considering the number of paths formed after different lag periods, the conditional configurations of each path are not listed.

The consistency of each path of the degree of industrial structure change is greater than 0.8, and the original coverage is greater than 0.5, indicating that these ten paths constitute sufficient conditions for the degree of change in the marine fishery industry structure. Comparing the consistency and coverage of each path in the first lag period, the second lag period, and the third lag period, the consistency and coverage of each path in the first period are the lowest, indicating that the market distortion of production factors in the marine fishery industry has a significant impact on the degree of industrial structure change. Lag is real.

Comparing the four paths of $\Delta S\_Moore_{t+1}$ during the phase 1 lag, the phase 2 lag, and the phase 3 lag under the impact of factor distortion, it can be found that the high factor distortion degree is not the core condition of the path; that is, the high factor market distortion will not lead to a high degree of change in the marine fishery industry. Among the six paths of $\sim \Delta S\_Moore_{t+1}$, DisK is the core condition; that is, when the capital factor is highly distorted, the marine fishery industry structure is less likely to undergo high-level changes. In each lag period, *DisL* and *DisR* act as marginal conditions at the same time in most cases or are missing at the same time, indicating that low labor factor distortion and low marine fishery resource factor distortion are more likely to inhibit high-level changes in the marine fishery industry structure and, at the same time, proving that *DisL* and *DisR* have a joint effect on the change of marine fishery industry structure.

The consistency of each path forming the high industrial structure upgrading index and the low industrial structure upgrading index is greater than 0.8, after subdividing the paths that can be expressed in the same way. The coverage of the industrial structure upgrading index under different lag periods is greater than 0.5, which shows that the original 11 paths can constitute the sufficient conditions for the upgrading of the marine fishery industrial structure. It can be seen that the consistency and coverage of each path in the first phase are the lowest, and the lag in the impact of the market distortion of the production factors of the marine fishery industry on the industrial structure upgrade index really exists.

Comparing the four paths of SU_t in lag phase I, lag phase II, and lag phase III under the influence of factor distortion, we can find that *DisL* and *DisK* are the core conditions; that is, high labor factor distortion and high capital factor distortion accelerate the upgrading of marine fishery industry structure. In the case of factor distortion affecting the second lag period, there are two completely opposite paths: “DisL*DisK*DisR” and “~DisL*~DisK* *~DisR*”, indicating that when the factor distortion of labor, capital, and fishery resources is low, the structure of the marine fishery industry is also prone to upgrade. We can conclude from the four paths of SU_t that the stronger the consistency of the distortion degree of marine fishery industry elements (that is, when the distortion is low or high at the same time), the faster the industrial structure upgrade speed. The conditional configuration of $\sim SU_{t}$ is relatively complex, and it can be further generalized into two main configurations on the basis of the obtained pathway: “~DisK*-” and “~DisL* *~DisR*”. Under the circumstance of low capital factor distortion, when either the market distortion degree of labor factor or marine fishery resource factor is “ high”, the rapid upgrading of marine fishery industrial structure is suppressed. During lag phase II or lag phase III, under the combination of the low labor factor distortion and the low distortion of marine fishery resources, the rapid upgrading of the marine fishery industrial structure is restrained and is not affected by the distortion of capital factors.

## 4. Explanations Based on Empirical Analysis

The fsQCA method has effectively identified multiple paths affecting the structural adjustment of the marine fishery industry, indicating that the factors that affect the structural adjustment of the marine fishery industry have the same outcome and multiple concurrencies [21]. Among them, each path to accelerating the structural upgrading of the marine fishery industry can be uniformly summarized as the mode of “highly consistent distortion of the factor market”; that is, when all factors are highly distorted or lowly distorted at the same time, the faster the upgrading of the marine fishery industry structure will be. The situation has played the role of “reversely forcing upgrade” and “promoting upgrade.” The paths to accelerate the upgrading of the marine fishery industry structure can be summarized into two modes: the “one low and one high” mode and the “two lows” lag mode. The former refers to the distortion of the low capital factor and high labor factor or the high marine fishery resources factor. The latter refers to the combined effect of low labor factor distortion and low marine fishery resource factor distortion. In general, capital factors play a crucial role in the process of upgrading the marine fishery industry structure, and there is a joint effect between labor and fishery resources.

### 4.1. Influence of Capital Factors and Its Status Quo of Distortion

At the macro level, China’s capital market has the typical characteristics of financial repression [32]; at the micro level, improving capital allocation efficiency or eliminating capital market distortions can significantly increase the total factor productivity [13,33]. The development of any industry is inseparable from the investment of capital. The investment in fixed assets of fisheries increases year by year, but the main source is self-raised funds and other funds. The dependence of fishery capital on domestic loans is very low, and it is declining year by year. For a long time, the source of funds for state-owned fishery enterprises has mainly relied on state-owned banks, while collective and private fishery enterprises have borrowed from commercial banks and credit cooperatives. The difficulty of obtaining fishery credit capital between state-owned and private fishery enterprises is different, and this has caused the price distortion of fishery capital to a certain extent. China’s financial system also has strict controls on the fishery capital market, such as restrictions on the establishment of banks by private fishery enterprises and control of the scale of fishery credit which, to a certain extent, promotes the distortion of fishery capital prices.

The root of the problem in the marine fishery industry is that fish is a public resource. If there is no economic incentive to ensure that the value of the fish resource is not compromised, then fishermen will overfish. In order to solve this problem and to improve the fishery industry structure at the same time, the government has increased its capital investment in fisheries, and it has successively revised and promulgated the *Fisheries Law of the People’s Republic of China*, *Fishery Development Plan* and *Development Plan for Aquaculture Area for Exporting Aquatic Product*. At the same time, after joining the WTO, the total amount of subsidies for the fishery industry has been continuously increased. However, compared with the subsidies for the farming industry, the number of fishery subsidies is still relatively small, and the total input is insufficient. In addition, the types of fishery subsidies are not rich enough to meet the actual needs of fishermen, and the types of indirect subsidies are more than those of direct subsidies. Therefore, the current investment of fishery subsidies cannot meet the status quo of marine fishery development. This is not beneficial to the upgrading of the fishery industry’s structure. Compared with other industries, fisheries are a high-risk industry. As offshore operations are affected by uncertain factors such as weather, it is difficult to form economies of scale, and the gross profit of the fishery industry is relatively low. The government’s innovative requirements for fisheries and the continuous attack and rejection of distant-water fisheries by social capital, bank capital, and securities capital have made the contradiction between supply and demand of funds in the development of fisheries increasingly prominent.

### 4.2. Influence of Labor Factors and Its Status Quo of Distortion

Under the ideal conditions of a perfectly competitive market, the equilibrium wage of workers is determined by the marginal productivity of labor, but in the real economy, the two may be unequal, and the labor market will experience market distortions that deviate from the ideal state. In the case of market segmentation, there is a phenomenon that labor cannot flow freely, resulting in different wages for the same labor in the market, and even high-quality labor cannot obtain matching wages in the market [34]. It also has a situation of labor market distortion. When workers are in a weak position due to information asymmetry or an imperfect labor market, the labor market will have negative distortions in which workers’ wages are lower than the equilibrium level; this negative distortion is also the main cause of the macroeconomic imbalance [35]. The government’s distorted policy of artificially separating the urban and rural labor markets is also an important reason for the growing income gap [36].

In recent years, the number of fishery practitioners has gradually decreased, some traditional marine fishermen changed their profession and turned to other related industries [37]. First, due to overfishing, the state has called for fishery practitioners to switch to other industries. Second, under the market economy system, the level of mechanization in the development of the fishery industry has increased, and labor is being deployed by the market to other industries. Among the fishery employees, the number of people engaged in marine fisheries accounts for 28.21%, of which the number of personnel engaged in marine fishing and marine aquaculture accounts for 82.30% of the professional employees in marine fisheries, and the proportion of the labor force engaged in the secondary and tertiary industries only accounts for 17.70% of the fishery professional practitioners. From the perspective of educational background, due to the particularity and history of marine fishery occupations, practitioners, especially fishery laborers engaged in marine fishing, generally have a low level of education, and it is difficult to improve this significantly in a short time [38]. In response to the low overall education level of the marine fishery labor force, although the Ministry of Agriculture has given a series of access conditions for marine fishery employment, the following problems still exist in specific production: the number of crew members does not match the number of existing fishing vessels; since the working staff on the boat is generally poorly educated and the situation cannot be significantly improved for a while, it is difficult for them to be promoted to positions of real professional crew members, and many of them work without qualifications or with false qualifications. Because the contribution rate of the fishery wage income to the total family income is low, many well educated young people are choosing industries with higher wages rather than returning to fishing where wages are lower.

### 4.3. The Influence and Distortion of Fishery Resource Elements

The output of marine products is mainly composed of the output of marine fishing and the output of mariculture. Before 2006, the output of marine fishing was always higher than the output of mariculture. In 2006, the output of mariculture in China exceeded the output of marine fishing for the first time. Since then, mariculture production has always been higher than marine capture production, and the gap has widened.

While fishery production has achieved rapid development, the marine ecological environment and fishery resources have also been severely damaged. The number and the power of fishing boats have increased, but the economic benefits have been declining. Traditional economic fish resources have been continuously destroyed, and the phenomenon of substitution between fish populations has become increasingly serious. High-quality traditional catches are replaced by low-quality secondary catches, and the ratio of the two types of catches has reversed. The distribution density of the original commercial fish stocks has also changed a lot, and the decline of fish resources in the middle and low layers as well as near the bottom layer is the most serious. In addition, the destruction of the living environment leads to the decline in the reproductive capacity of fishery resources, mainly due to the discharge of land-based pollutants, ship pollution, and unreasonable construction in marine engineering [39].

In response to the destruction of the marine ecological environment, the sharp drop in the output of aquatic products, and the serious decline of fishery resources, the government has successively introduced relevant policies such as “dual control”, “value-added release”, “fishing suspension during seasonal periods”, “zero growth”, “reduction of boats”, “marine protected areas”, and “marine pastures”, which have alleviated the problem of the decline of fishery resources to a certain extent. They have also played a regulating role in the marine ecological environment, but almost every policy has shortcomings, and the contradiction between limited fishery resources and overfishing has not been truly resolved.

There is an interactive relationship between factor prices and industrial structure [40]. The distortion of factor markets has a significant inhibitory effect on the upgrading of industrial structure, and the effects of price distortions of labor, capital, resources, and other factors are different [41]. In the marine fishery industry, labor market distortion has a greater impact than capital and resource market distortion [42]. A distorted factor market leads to a distorted factor price relationship. The input remains unchanged. If the distortion of the factor market is eliminated, the total output will increase significantly [43]. The supply structure reform of the marine fishery is one of the sustainable development trends of marine fisheries [44].

## 5. Discussions and Implementations

Adjusting the distortion degree of the factor market to optimize the marine fishery industry structure is an effective measure in reducing the vulnerability of China’s marine fishery industry ecosystem. Reversing the negative relationship between fishermen’s professional level and efficiency will help optimize the marine fishery industry structure and will enhance the industrial economic value. Therefore, this study puts forward the following three suggestions for the development of the marine fishery industry.

### 5.1. Improve the Quality of Labor Factors

According to the 2020 China Fishery Statistics Yearbook, about 88.23% of the fishery workers are engaged in the primary industry of fishing and aquaculture, and only 11.77% of the fishery professionals are engaged in secondary and tertiary industries. From the perspective of educational structure, the educational level of fishery workers is low, especially among the fishery labor force engaged in marine fishing. There are few crew members who have received both high school and vocational high school education or above. Therefore, one of the prerequisites for the rational allocation of labor force elements is to improve the education level of fishery employees, thus making them able to adapt to modern fisheries and to transform from traditional fisheries to modern fisheries so as to promote the adjustment of the fishery industrial structure from the primary industry to the secondary and tertiary industries.

### 5.2. Reasonably Allocate Capital Elements

The capital allocation in the fishery market is not balanced. The funds needed by enterprises generally come from loans. Most enterprises borrow from banks. Because banks are worried about risks, they will increase financing for enterprises with stable returns and low risks, and they will be cautious in financing high-leverage and high-risk companies. However, more often than not, such highly leveraged, high-risk businesses can generate higher productivity, which can lead to capital mismatches. Therefore, the rational allocation of capital elements and increased support for secondary and tertiary industry enterprises are also particularly important for the optimization of industrial structure.

### 5.3. Strengthen the Management of Marine Resource Elements

Many countries are rich in marine fishery resources. The pelagic fishery model, which is restricted by factors such as its management system, industrial scale, and lack of effective fishery policy guarantee, currently accounts for a small proportion of the entire fishery industry, so there is still a lot of room for development. It is of great significance to increase investment in scientific research and development in order to promote scientific and technological support for fishery development and to use advanced technology to improve the development and utilization of fishery resources. The core concept of Industry 4.0 is to integrate advanced information technologies [45]. The Internet of Things (IoT) and blockchain have made great progress [46,47], and 5G or 6G will present us with the ability to establish full coverage of the “air–space–sea–land” system and to integrate [48]. All the above technologies related to quantum computers are potential for improving and changing the management of marine resource elements in many aspects [49]. At the same time, the elements of marine resources and recreational fisheries can be integrated to further optimize the structure of the fishery industry and to promote the sustainable development of marine fisheries.

## 6. Conclusions, Limitations, and Implications

### 6.1. Conclusions

We focused on the marine environment and sustainable development of the fishery industry in this study. By reviewing the research on the distortion of labor, capital, and technology factors and by combining the development and the upgrading status of the marine fishery industry, we used industry macro data to measure the distortion of market factor prices. We built a Moore-like index and a simplified industrial structure upgrading index. Moreover, based on the qualitative comparative analysis of fsQCA fuzzy set, we further studied the impact of factor market distortion on the industrial structure of fisheries.

### 6.2. Limitations

In this paper, the marginal role of each factor in the development of the marine fishery industry, the causes of distortion, and the deeper impact mechanism, including the interaction between factors, need further study. Future research can be carried out from the following aspects: (1) theoretically and empirically analyze the marginal role of each factor in the different stages of the development of the marine fishery industry; (2) further summarize the causes of distortion in the factor market; (3) study the interaction between various elements; (4) try to further improve the comprehensive evaluation method and index system, and more accurately measure the distortion level of the factor market and the characteristics of the optimization and upgrading of the marine fishery industry.

### 6.3. Implications

In combination with the current situation of the development of the marine fishery industry and the degree of distortion in the factor market price, we put forward the following suggestions from the aspects of labor factors, capital factors, fishery resources factors, industrial structure, and other aspects: (1) improve the quality of labor factors, improve the violation cost of fishery operators, conduct regular training for fishery practitioners, and establish a complete crew wage guarantee system; (2) strengthen the management of marine resources’ elements, refine the regional division system of fishery resources, regulate the catch after the end of the fishing moratorium, and improve the utilization rate of fishery resources; (3) rationally allocate capital elements, increase the total amount of subsidies to fisheries, and broaden the financing channels for fisheries; (4) learn from foreign experience to further optimize the industrial structure, vigorously develop recreational fisheries, and improve scientific and technological productivity and specialization.

## Figures and Tables

**Table 1:** Price Distortions of Various Factors in the Marine Fishery Industry from 1999 to 2019.

Year	*DisL*	*DisK*	*DisR*
1999	0.6877	258.0876	0.4688
2000	0.6462	208.5443	0.4840
2001	0.5053	213.0883	0.4668
2002	0.5358	178.1359	0.5043
2003	0.2821	108.9391	0.4593
2004	0.2731	48.7149	0.4374
2005	0.2690	43.0393	0.4523
2006	0.2498	39.7871	0.5070
2007	0.1244	39.2617	0.4328
2008	0.2267	179.9414	0.2642
2009	0.3913	170.7733	0.7366
2010	0.2883	126.6691	0.4338
2011	0.2361	69.3258	0.4171
2012	0.2660	57.8301	0.4430
2013	0.2736	54.0333	0.4630
2014	0.2873	50.8854	0.4768
2015	0.4217	52.4845	0.4832
2016	1.3962	54.6509	0.4789
2017	1.3980	51.5971	0.4564
2018	1.500	43.3481	0.5110
2019	1.4808	40.2320	0.4908

Note: The factor output elasticity from 1999 to 2019 is considered constant.

**Table 2:** Indicators of Changes in Fishery Industrial Structure.

Year	$S\_Moore_{t+1}^{1}$	$S\_Moore_{t+1}^{2}$	$S\_Moore_{t+1}^{3}$	$\Delta S\_Moore_{t+1}$	$SU_{t}$
2001	0.1971	0.7873	0.8539	0.7496	0.0645
2002	0.2290	0.7771	0.8310	0.8326	0.0335
2003	0.2732	0.7633	0.7990	0.8799	0.0981
2004	0.3112	0.7588	0.7672	0.9857	0.0178
2005	0.3274	0.7546	0.7554	1.0031	0.0283
2006	0.3485	0.7426	0.7470	1.0325	0.0194
2007	0.3667	0.7307	0.7413	1.0534	0.0190
2008	0.3678	0.7255	0.7454	1.0731	−0.0243
2009	0.3683	0.7320	0.7384	1.0494	0.0366
2010	0.5391	0.7365	0.5321	1.0189	−0.0116
2011	0.7332	0.7359	0.3697	0.9995	−0.0026
2012	0.7330	0.7364	0.3694	0.9968	0.0024
2013	0.7302	0.7373	0.3714	0.9993	0.0055
2014	0.7263	0.7400	0.3727	0.9980	0.0029
2015	0.5389	0.7413	0.5295	1.0173	0.0147
2016	0.3917	0.7419	0.7058	1.0829	0.0308
2017	0.4028	0.7434	0.6937	1.1062	0.0061
2018	0.4046	0.7515	0.6835	1.1067	0.0131
2019	0.4094	0.7548	0.6754	1.1117	0.0074

**Table 3:** Necessity Analysis Table (Degree Value of Industrial Structure Change).

	$\Delta S\_Moore_{t+1}^{T=1}$	$\sim \Delta S\_Moore_{t+1}^{T=1}$	$\Delta S\_Moore_{t+1}^{T=2}$	$\sim \Delta S\_Moore_{t+1}^{T=2}$	$\Delta S\_Moore_{t+1}^{T=3}$	$\sim \Delta S\_Moore_{t+1}^{T=3}$
*DisL*	0.6597	0.6639	0.6257	0.6944	0.6125	0.6806
*~DisL*	0.6855	0.7588	0.7161	0.7506	0.6718	0.6477
*DisK*	0.5306	0.8513	0.4951	0.8849	0.4490	0.8657
*~DisK*	0.7562	0.5000	0.7593	0.4463	0.7794	0.3980
*DisR*	0.6291	0.6616	0.6375	0.6586	0.6422	0.6172
*~DisR*	0.6119	0.6335	0.6277	0.6867	0.6279	0.6946

Note: $\Delta S\_Moore_{t+1}^{T=1},\;\Delta S\_Moore_{t+1}^{T=2},\;\Delta S\_Moore_{t+1}^{T=3}\;$ represent the values of the industrial structure change degree in lag phase I, lag phase II, and lag phase III under the influence of factor distortion. The same below.

**Table 4:** Necessity Analysis Table (Industrial Structure Upgrading Indicators).

	$SU_{t}^{T=1}$	$\sim SU_{t}^{T=1}$	$SU_{t}^{T=2}$	$\sim SU_{t}^{T=2}$	$SU_{t}^{T=3}$	$\sim SU_{t}^{T=3}$
*DisL*	0.6797	0.5996	0.6633	0.5965	0.7627	0.6089
*~DisL*	0.6719	0.7141	0.6757	0.7336	0.6385	0.7520
*DisK*	0.7377	0.6145	0.6892	0.6930	0.7354	0.6436
*~DisK*	0.5926	0.6803	0.6565	0.6436	0.6174	0.6737
*DisR*	0.7377	0.6195	0.6914	0.6601	0.7702	0.6011
*~DisR*	0.6105	0.6912	0.6712	0.6930	0.6422	0.6011

Note: $\;SU_{t}^{T=1},\;SU_{t}^{T=2},\;SU_{t}^{T=3}$ represent the industrial structure upgrading values of lag phase I, lag phase II, and lag phase III under the influence of factor distortion, respectively. The same below.

**Table 5:** Configuration of Factors Influencing the Change Degree of Marine Fishery Industrial Structure.

	$\Delta S\_Moore_{t+1}^{T=1}$	$\sim \Delta S\_Moore_{t+1}^{T=1}$		$\Delta S\_Moore_{t+1}^{T=2}$	$\sim \Delta S\_Moore_{t+1}^{T=2}$		$\Delta S\_Moore_{t+1}^{T=3}$		$\sim \Delta S\_Moore_{t+1}^{T=3}$	
*DisL*	⨂	⊗	•	-	⊗	•	⊗	-	⊗	•
*DisK*	-	⬤	⬤	⨂	⬤	⬤	⨂	⨂	⬤	⬤
*DisR*	-	⊗	•	-	⊗	•	-	•	⊗	⬤
*Consistency*	0.8617	0.8495	0.7922	0.8957	0.8382	0.8046	0.8738	0.9774	0.7996	0.7817
*Raw coverage*	0.7562	0.5222	0.5222	0.7593	0.5563	0.5793	0.5851	0.5686	0.4753	0.5311
*Unique coverage*	0.7562	0.2119	0.2119	0.7593	0.2020	0.2251	0.1372	0.1207	0.2091	0.2649
*Solution consistency*	0.8617	0.7731		0.8957	0.7813		0.8894		0.8111	
*Solution coverage*	0.7562	0.7342		0.7593	0.7997		0.7058		0.7402	

Note: ⬤ represents the occurrence of core conditions; • represents the existence of marginal conditions; ⨂ represents the absence of core conditions; ⊗ represents the absence of marginal conditions; - represents that the variable was not reported by the result.

**Table 6:** Configuration of Influencing Factors for Upgrading Indicators of Marine Fishery Industrial Structure.

	$SU_{t}^{T=1}$	$\sim SU_{t}^{T=1}$		$SU_{t}^{T=2}$		$\sim SU_{t}^{T=2}$			$SU_{t}^{T=3}$	$\sim SU_{t}^{T=3}$	
*DisL*	⬤	-	⬤	⨂	•	⬤	-	⊗	⬤	⨂	⬤
*DisK*	•	⊗	⨂	⨂	•	⨂	⨂	⬤	•	-	⨂
*DisR*	•	⊗	-	⨂	•	-	⬤	⊗	-	⨂	•
*Consistency*	0.8419	0.8620	0.8654	0.8880	0.8863	0.8825	0.8516	0.8131	0.8540	0.8375	0.8712
*Raw coverage*	0.5290	0.5100	0.4612	0.5000	0.5619	0.4693	0.5285	0.4627	0.6683	0.6793	0.4156
*Unique coverage*	0.5290	0.1783	0.1295	0.2432	0.3052	0.0395	0.1031	0.2105	0.6683	0.3799	0.1162
*Solution consistency*	0.8419	0.8210		0.8667		0.8163			0.8540	0.8386	
*Solution coverage*	0.5290	0.6394		0.8052		0.7895			0.6683	0.7955	

Note: ⬤ represents the occurrence of core conditions; • represents the existence of marginal conditions; ⨂ represents the absence of core conditions; ⊗ represents the absence of marginal conditions; - represents that the variable was not reported by the result.

## Data Availability

The data supporting reported results can be found at: https://fishery.ckcest.cn/featureYearbook.html (accessed on 23 May 2022).
